# Gamma Knife Radiosurgery for Brain Metastases in Non-Small Cell Lung Cancer Patients Treated with Immunotherapy or Targeted Therapy

**DOI:** 10.3390/cancers12123668

**Published:** 2020-12-07

**Authors:** Anna Cho, Helena Untersteiner, Dorian Hirschmann, Abdallah Shaltout, Philipp Göbl, Christian Dorfer, Karl Rössler, Wolfgang Marik, Klaus Kirchbacher, Irene Kapfhammer, Sabine Zöchbauer-Müller, Brigitte Gatterbauer, Maximilian J. Hochmair, Josa M. Frischer

**Affiliations:** 1 Department of Neurosurgery, Medical University of Vienna, 1090 Vienna, Austria; anna.cho@meduniwien.ac.at (A.C.); n1542504@students.meduniwien.ac.at (H.U.); dorian.hirschmann@meduniwien.ac.at (D.H.); abdallah.shaltout@hotmail.com (A.S.); philipp.goebl@hotmail.com (P.G.); christian.dorfer@meduniwien.ac.at (C.D.); karl.roessler@meduniwien.ac.at (K.R.); brigitte.gatterbauer@meduniwien.ac.at (B.G.); 2 Department of Biomedical Imaging and Image-guided Therapy, Division of Neuroradiology and Musculoskeletal Radiology, Medical University Vienna, 1090 Vienna, Austria; wolfgang.marik@meduniwien.ac.at; 3 Department of Pulmology, Wilhelminenspital, 1160 Vienna, Austria; klaus.kirchbacher@wienkav.at; 4 Pulmology Department, Otto Wagner Spital, 1140 Vienna, Austria; irene.kapfhammer@wienkav.at; 5 Department of Internal Medicine I, Division of Oncology, Medical University Vienna, 1090 Vienna, Austria; sabine.zoechbauer-mueller@meduniwien.ac.at; 6 Department of Respiratory and Critical Care Medicine, Karl Landsteiner Institute of Lung Research and Pulmonary Oncology, Vienna North Hospital, 1210 Vienna, Austria; maximilian.hochmair@wienkav.at

**Keywords:** Gamma Knife radiosurgery, immunotherapy, targeted therapy, lung cancer, brain metastases

## Abstract

**Simple Summary:**

In non-small cell lung cancer patients with brain metastases, combined Gamma Knife radiosurgery and immunotherapy or targeted therapy showed an increase in overall survival. The combination of Gamma Knife radiosurgery and immunotherapy or targeted therapy did not increase complications related to radiosurgery. Therefore, the combined treatment seems to be a safe and powerful treatment option for non-small cell lung cancer patients with brain metastases.

**Abstract:**

The combination of Gamma Knife radiosurgery (GKRS) and systemic immunotherapy (IT) or targeted therapy (TT) is a novel treatment method for brain metastases (BMs) in non-small cell lung cancer (NSCLC). To elucidate the safety and efficacy of concomitant IT or TT on the outcome after GKRS, 496 NSCLC patients with BMs, who were treated with GKRS were retrospectively reviewed. The median time between the initial lung cancer diagnosis and the diagnosis of brain metastases was one month. The survival after the initial BM diagnosis was significantly longer than the survival predicted by prognostic BM scores. After the first Gamma Knife radiosurgery treatment (GKRS1), the estimated median survival was 9.9 months (95% CI = 8.3–11.4). Patients with concurrent IT or TT presented with a significantly longer survival after GKRS1 than patients without IT or TT (*p* < 0.001). These significant differences in the survival were also apparent among the four treatment groups and remained significant after adjustment for Karnofsky performance status scale (KPS), recursive partitioning analysis (RPA) class, sex, and multiple BMs. About half of all our patients (46%) developed new distant BMs after GKRS1. Of note, no statistically significant differences in the occurrence of radiation reaction, radiation necrosis, or intralesional hemorrhage in association with IT or TT at or after GKRS1 were observed. In NSCLC-BM patients, the concomitant use of GKRS and IT or TT showed an increase in overall survival without increased complications related to GKRS. Therefore, the combined treatment with GKRS and IT or TT seems to be a safe and powerful treatment option and emphasizes the role of radiosurgery in modern BM treatment.

## 1. Introduction

In non-small cell lung cancer (NSCLC) patients, brain metastases (BMs) occur in up to 60% of patients, representing the most common cause of BMs [1]. Depending on the number and location of the BMs, surgery, stereotactic radiosurgery (SRS), and whole-brain irradiation (WBRT) are used as local treatments. So far, only a few systemic therapies, if indeed, might penetrate the blood-brain barrier [1,2]. As compared with WBRT, Gamma Knife radiosurgery (GKRS) enables the delivery of a high radiation dose to the selected target with a rapid radiation fall-off to the surrounding brain parenchyma. Consequently, high tumor control rates are achieved with almost no neurocognitive deterioration [3,4]. A novel treatment method for BMs in NSCLC patients is the combination of GKRS and immunotherapy (IT) or targeted therapy (TT) [5]. Recent preliminary studies have suggested that this new form of combination therapy might amplify the immune response to malignant cells, and thereby improve the overall survival of patients with BMs [6,7]. So far, only inadequate data on the safety and efficacy of the concomitant use of SRS and systemic treatment with either IT or TT exist. The aim of our study was to investigate the safety and efficacy profile of this combination treatment for NSCLC patients with BMs.

## 2. Results

### 2.1. Patient Characteristics and Overall Follow-Up

Detailed patient characteristics including the prognostic scores are displayed in Table 1. The median time between the initial lung cancer diagnosis and the diagnosis of BMs was 1.0 month (0.0–216.6). At brain metastasis (BM) diagnosis, 321/496 (65%) of patients had already been diagnosed with extracranial metastases. Consequently, the majority of patients (372/496, 75%) were rated as recursive partitioning analysis (RPA) class II. 

After the initial BM diagnosis, the median follow-up period was 10.7 (0.4–113.9) months. The estimated median overall survival was 19.6 months (95% confidence interval (CI) = 16.8–22.5) after the initial diagnosis of NSCLC, 12.5 months (95% CI = 10.8–14.1) after the initial diagnosis of BMs, and 9.9 months (95% CI = 8.3–11.4) after the first Gamma Knife treatment (GKRS1).

Patients with RPA class I (32.2 months, 95% CI = 21.0–43.5) had the longest survival after GKRS1, followed by patients with RPA class II (8.8 months, 95% CI = 7.2–10.3) and III (2.0 months, 95% CI = 0.4–3.6, *p* < 0.001). 

Patients with a Karnofsky performance status scale (KPS) of 80% or above (13.8 months, 95% CI = 11.4–16.2) showed a longer survival after GKRS1 as compared with patients with a KPS below 80% (3.1 months, 95% CI = 2.1–4.1, *p* < 0.001). Additionally, the survival was compared with the survival predicted from the prognostic scores. This subanalysis showed that our patients had a significantly longer survival after GKRS as compared with the survival predicted by the general graded prognostic assessment (GPA, *p* < 0.001), specific GPA (*p* < 0.001), RPA (*p* < 0.001), and the Score Index for Radiosurgery (SIR; *p* < 0.001). The survival rates after GKRS1 were 62% at six months, 44% at 12 months, and 18% at 36 months, respectively.

**Figure 1:**
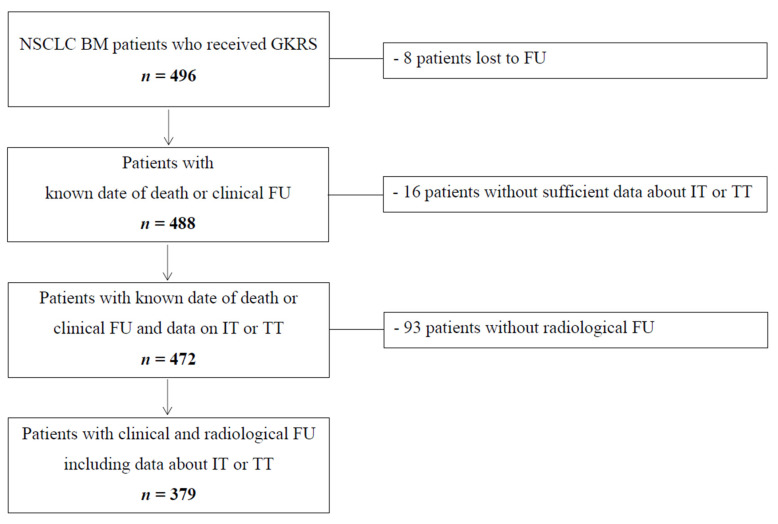
Flow chart depicting study inclusion algorithm. BM, brain metastasis, GKRS, Gamma Knife radiosurgery; FU, follow-up; IT, immunotherapy; MRI, magnetic resonance imaging; NSCLC, non-small cell lung cancer; TT, targeted therapy.

Table 1 depicts patient sample characterization at the time of first GKRS for the total sample and for follow-up patients with or without IT or TT treatment at or after the first GKRS treatment (GKRS1). As described in Figure 1, 8/496 (2%) patients were lost to follow-up and a further 16/496 (3%) patients did not have any data on IT or TT at or after GKRS (*). After excluding these patients, the baseline characteristics were additionally evaluated for 186/472 (39%) patients in the “IT or TT group” (defined as IT or TT or a combination of IT and TT) and for 286/472 (61%) patients in the “none group” (defined as no IT or TT). 

Prior CNS treatment was mainly performed for distant brain metastases. We evaluated the graded prognostic assessment (GPA general and specific), the updated graded prognostic assessment for lung cancer using molecular markers (Lung-molGPA), recursive partitioning analysis (RPA), and the Score Index for Radiosurgery (SIR) for each patient. Gamma Knife parameters are given for the first radiosurgery treatment.

Between 2012 and 2018, 496 NSCLC patients with BMs underwent Gamma Knife radiosurgery treatment. According to our clinical standard protocol, patients were followed in a three-month intervals with a clinical assessment and a brain MRI. However, as known in everyday clinical practice, patients did not always keep their scheduled appointments. In addition, a death register comparison for all patients was performed. Patients, who were lost to follow-up (FU), were included in the study but excluded from the outcome analysis. At the time of study conclusion, only 8/496 (2%) patients were truly lost to follow-up. Therefore, 488/496 (98%) patients were included for the survival outcome analyses. At the conclusion of the study, a total of 372/488 (76%) patients had died. After excluding patients without sufficient data about IT or TT (16/488, 3%), 472 patients were available for the survival analyses. Of those, patients without radiological FU were excluded from the complication analysis, resulting in a total of 379 patients. 

### 2.2. Overall Outcome and Complications after GKRS and Concurrent Immunotheraphy (IT) or Targeted Therapy (TT)

**Figure 2:**
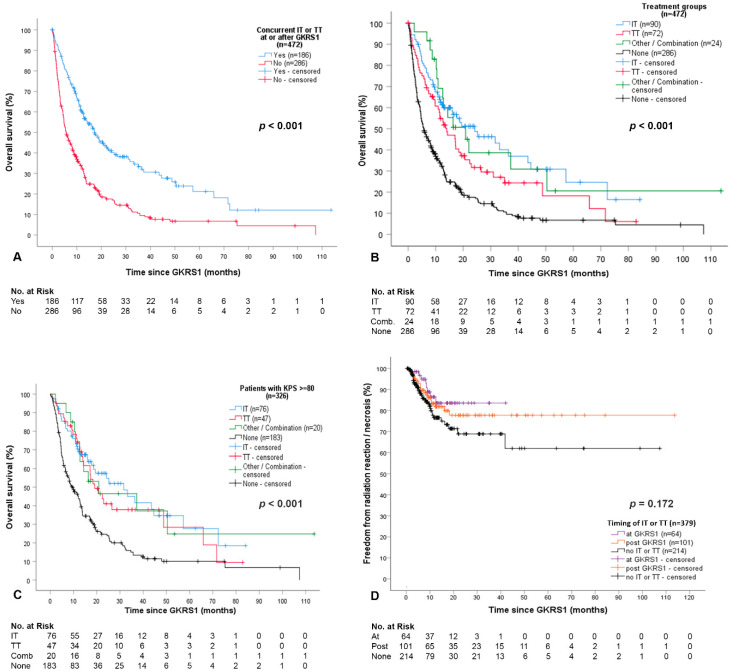
Differences of survival and overall complications between the treatment groups. (**A**) Survival after GKRS1 was significantly longer in patients treated with concurrent IT or TT or a combination of IT and TT at or after GKRS1 (186/472 or 39%,17.2 months, 95% CI = 12.6–21.7) than in patients without IT or TT (286/472 or 61%, 5.6 months, 95% CI = 4.2–7.0, *p* < 0.001); (**B**) The estimated median survival time was 24.2 months (95% CI = 10.7–37.8) in the IT group, 14.0 months (95% CI = 8.7–19.3) in the TT group, 20.9 months (95% CI = 11.1–30.7) in the combination group, and 5.6 months (95% CI = 4.2–7.0) in patients without any IT or TT (*p* < 0.001). Pairwise comparisons between each treatment group versus no IT or TT showed a statistically significant longer survival as compared with the “none” group even after correction for multiple testing (*p* < 0.001, *p* < 0.001, *p* < 0.001); (**C**) To minimize any bias from patients with poor functional impairment and prognosis before treatment, the same analysis was performed in respect of the Karnofsky performance status scale (KPS) and recursive partitioning analysis (RPA) classifications. The survival after GKRS1 among the four treatment groups remained statistically significant after including only patients with a minimum KPS of 80 at GKRS1 (326/472 or 69%, *p* < 0.001). The estimated median survival time was 31.7 months (95% CI = 16.7–46.6) in the IT group, 19.1 months (95% CI = 13.0–25.2) in the TT group, 20.9 months (95% CI = 0.0–45.7) in the combination group, and 9.6 months (95% CI = 6.5–12.8) in patients without any IT or TT. Pairwise comparisons between each treatment group versus no IT or TT showed a statistically significant longer survival after GKRS1 as compared with the “none” group even after correction for multiple testing (*p* < 0.001, *p* = 0.002, *p* = 0.008); (**D**) The estimated median time to occurrence of radiation reaction and necrosis were compared between patients with IT or TT at or after GKRS, and patients without any IT or TT. This comparison was not found to be statistically different (*p* = 0.172). Pairwise tests between the at and after GKRS groups (*p* = 0.428), at GKRS and none groups (*p* = 0.085), as well as after GKRS and none groups (*p* = 0.268) also did not reveal significant differences. These results remained the same even when radiation necrosis (*p* = 0.501) and radiation reaction (*p* = 0.046 and *p* = 0.139 after adjustment for multiple testing) were evaluated separately. CI, confidence interval; GKRS, Gamma Knife radiosurgery; IT, immunotherapy; KPS, Karnofsky Performance Status Scale; TT, targeted therapy.

Details on IT or TT are presented in Table 2. Patients with IT or TT had a significantly longer survival after GKRS1 than patients without IT or TT (*p* < 0.001, Figure 2A), even when separated into the four treatment groups (*p* < 0.001, Figure 2B). 

Moreover, survival after GKRS1 was significantly longer in patients who received IT or TT at GKRS (82/472 or 17%, 12.7 months, 95% CI = 8.4–16.9, *p* = 0.004) and in patients who received IT or TT after GKRS (104/472 or 22%, 24.2 months, 95% CI = 12.1–36.4, *p* < 0.001) as compared with the none group (286/472 or 61%, 5.6 months, 95% CI = 4.2–7.0).

In our study, 8/496 (2%) NSCLC patients with BMs were lost to follow-up (Figure 1). Our follow-up patients were grouped into those who received immunotherapy (IT group, *n* = 90/488, 18%), targeted therapy (TT group, *n* = 72/488, 15%), a combination of IT and TT (combination group, *n* = 24/488, 5%), and those who were not treated with any form of IT or TT (none group, *n* = 286/488, 59%). In 16/488 patients (3%), no data on IT/TT was available.

To minimize any bias from patients with poor functional impairment and prognosis before treatment, we performed several subanalyses; even after including only those patients with a minimum KPS of 80% at GKRS1, differences in survival after GKRS1 remained statistically significant among the four treatment groups (*p* < 0.001, Figure 2C). 

Next, we performed the same analysis in respect to sex and number of BMs. Differences in survival after GKRS1 among the IT or TT subgroups remained statistically significant among female (*p* < 0.001) and male patients (*p* < 0.001) and among patients with single (*p* < 0.001) or multiple BMs (*p* < 0.001) at GKRS1. Among only those patients rated as RPA class II, the differences in survival also remained statistically significant between the four treatment groups (*p* < 0.001). 

Among patients with RPA classes I and III, this separate analysis was infeasible due to the small sample size. Therefore, patients were pooled into cohorts of patients with and without IT or TT treatment only for RPA classes I and III. In patients with RPA I, no significant differences could be observed between both groups (*p* = 0.216). In RPA class III, patients with concurrent IT or TT showed a longer survival after GKRS1 than in patients without IT or TT (*p* = 0.036). Survival in patients with IT or TT was comparable to the predicted survival calculated by the Lung-molGPA score but was significantly reduced in patients without IT or TT (*p* < 0.001). 

In our cohort, 84 patients (84/472, 18%) received salvage therapy, defined as either GKRS boost or microsurgical resection due to local progression, as well as WBRT due to multiple new BMs or even combinations thereof. The majority of patients that underwent salvage therapy received WBRT (65/84, 77%). The estimated median survival time after GKRS1 did not show any significant differences between patients with WBRT after GKRS1 (65/472 or 14%, 13.4 months, 95% CI = 10.5–16.3) and patients without WBRT (407/472 or 86%, 8.9 months, 95% CI = 6.9–10.8, *p* = 0.791). Similar results were seen when only analyzing patients in the IT or TT group.

Of note, the rates of hemorrhages, radiation reaction (RR), and radiation necrosis (RN) in relation to IT or TT treatments are depicted in Table 3. 

After excluding patients lost to FU, without sufficient data about IT or TT, or without clinical or radiological FU, a total of 379 patients could be evaluated for radiologically diagnosed complications. All radiologically diagnosed complications were considered, even in cases of asymptomatic patients. Overall, the occurrence of complications in association with IT or TT after GKRS1 did not differ among the treatment groups (hemorrhages Chi-square *p* = 0.671, radiation reaction Chi-square *p* = 0.436, radiation necrosis Chi-square *p* = 0.752). Additionally, pairwise comparisons of the occurrences of complications among the different groups were performed. The occurrence of hemorrhage after GKRS1 did not show any differences among patients with IT and without IT or TT *p* = 0.380), among patients with TT and without IT or TT (*p* = 0.456), and among patients with combined IT/TT and without IT or TT (*p* = 0.634). 

The occurrence of radiation reaction after GKRS1 did not show any differences among patients with IT and without IT or TT (*p* = 0.955), among patients with TT and without IT or TT (*p* = 0.190), and among patients with combined IT or TT and without IT or TT (*p* = 0.310). The occurrence of radiation necrosis after GKRS1 also did not show any differences among patients with IT and without IT or TT (*p* = 0.430), among patients with TT and without IT or TT (*p* = 0.598), and among patients with combined IT/TT and without IT or TT (*p* = 0.811). Additionally, direct comparisons of the IT versus the TT treatment group did not result in any significant difference in the occurrence of complications after GKRS1 either for radiation reaction (*p* = 0.216) or radiation necrosis (*p* = 0.308) alone or for the occurrence of pooled complications (*p* = 0.094). 

Overall, no statistically significant differences in the occurrence of complications in association with IT or TT after GKRS1 could be observed (hemorrhages *p* = 0.671, RR *p* = 0.436, RN *p* = 0.752). Additional pairwise comparisons also did not reveal any significant differences in the occurrence of hemorrhages, RR, or RN among the different treatment groups (Table 3). Furthermore, the estimated median time to occurrence of complications was not found to be statistically different among patients with IT or TT at or after GKRS, and patients without any IT or TT (Figure 2D). Of note, patients with IT or TT or a combination of IT and TT at GKRS1 did not show any local progression (0/64, 0%), while 11% (11/101) of patients with IT or TT or a combination therapy after GKRS1, and 8% (16/214) of patients in the none group it was seen (*p* = 0.029). In patients with IT or TT at GKRS1, PFS was significantly longer than in patients who received IT or TT after GKRS (*p* = 0.038), and also as compared with patients without IT or TT (*p* = 0.010).

To further elucidate the effect of IT or TT on the outcome after GKRS, we performed several further subanalyses. 

### 2.3. Detailed Outcome and Complications after GKRS with Concurrent Immunotherapy (IT Group) 

**Figure 3:**
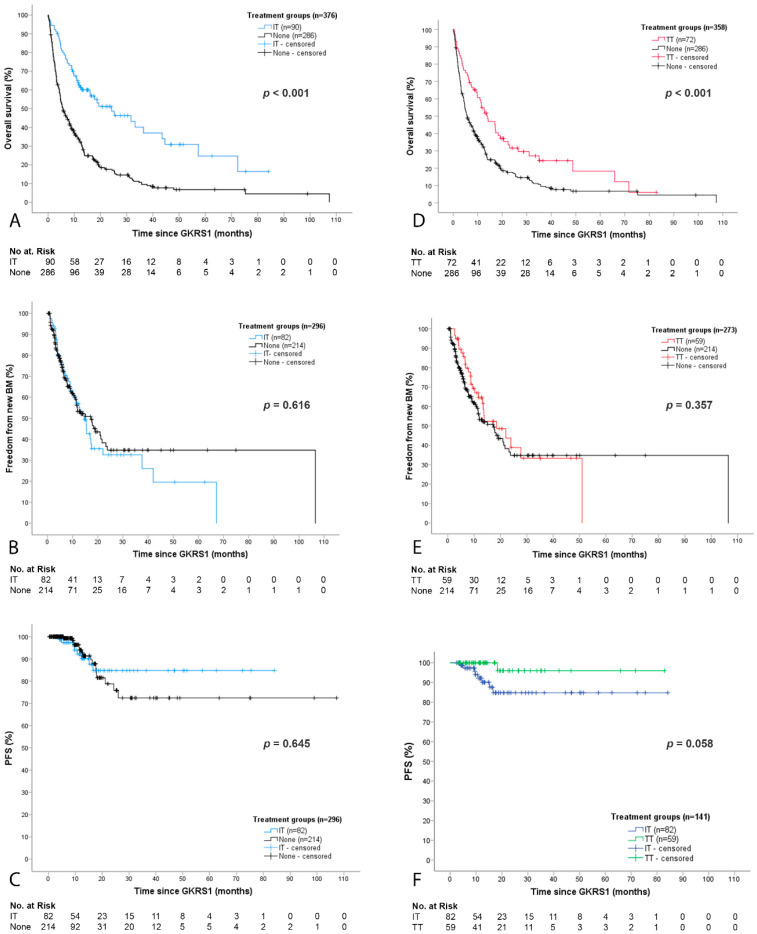
Outcome after GKRS1 separated for IT or TT. (**A**) Survival after GKRS1 was significantly longer in the IT group (24.2 months, 95% CI = 10.7–37.8) as compared with patients without any concurrent oncological therapies (5.6 months, 95% CI = 4.2–7.0, *p* < 0.001); (**B**) Radiological data on the occurrence of new BMs, local progression, and radiation reaction/necrosis were available for 82/90 (91%) patients with IT and 214/286 (75%) patients without IT or TT. The time to new brain metastases did not differ between the IT and the none group (*p* = 0.616); (**C**) The estimated mean local progression-free survival did not differ between the IT and the none group (*p* = 0.645); (**D**) The TT group showed a significantly longer estimated survival (14.0 months, 95% CI = 8.7–19.3) as compared with patients without IT or TT (5.6 months, 95% CI = 4.2–7.0, *p* < 0.001); (**E**) Radiological data and follow-up data were available for 59/72 (82%) patients with TT. The estimated median time to new brain metastases showed no significant differences in the TT group (18.5 months, 95% CI = 7.2–29.8) as compared with the none group (17.2 months, 95% CI = 11.7–22.8, *p* = 0.357); (**F**) The estimated mean time to local progression was longer in patients with TT (80.2 months, 95% CI = 75.3–85.2) as compared with patients in the IT group (73.1 months, 95% CI = 65.8–80.4, *p* = 0.029 and *p* = 0.058 after adjustment for multiple testing). BM, brain metastasis; CI, confidence interval; GKRS, Gamma Knife radiosurgery; IT, immunotherapy; TT, targeted therapy.

The estimated median survival was significantly higher in patients with IT as compared with patients without IT or TT (*p* < 0.001, Figure 3A). About half of all patients presented with new BMs after GKRS1 (IT group, 46/82 or 56% and the none group, 88/214 or 41%). However, no significant difference in the estimated median time to new BMs could be observed (*p* = 0.616, Figure 3B). 

At last follow-up, the vast majority of patients with IT (74/82 or 90%) and without IT or TT (196/214 or 92%) presented with stable or decreased BMs. The estimated time to progression after GKRS1 did not differ between the IT and the none group (*p* = 0.645, Figure 3C). Although a trend was seen, there was no statistically significant difference in the local PFS in patients with IT at GKRS1 (*n* = 20) as compared with patients who received IT after GKRS1 (*n* = 62).

The overall comparison of complication rates among the different treatment groups are described in Table 3. The occurrence of radiation reaction in patients who received concomitant IT at GKRS1 did not show any differences as compared with patients without IT or TT (*p* = 0.123) or patients whose IT was started > 30 days after GKRS (*p* = 0.414). 

In a similar fashion, the occurrence of radiation necrosis in patients, who received concomitant IT at GKRS1, did also not show any differences as compared with patients without IT or TT (*p* = 0.622) or patients whose IT was started >30 days after GKRS (*p* = 0.485). These analyses did not differ even when the occurrence of RR and RN were pooled (*p* = 0.378 at GKRS1 and *p* = 0.257 after GKRS1). 

The Kaplan–Meier estimated mean time to occurrence of RR alone, RN alone, and RR/RN pooled did not show any differences in patients, who started with IT at GKRS1 versus those patients who started IT after GKRS1 (*p* = 0.129 for RR, *p* = 0.629 for RN, and *p* = 0.456 for RR/RN pooled). 

### 2.4. Detailed Outcome and Complications after GKRS with Concurrent Targeted Therapy (TT Group)

The TT group showed a significantly longer estimated survival after GKRS1 (*p* < 0.001, Figure 3D). Half of all patients presented with new BMs after GKRS1 in the group as well (TT group, 27/59 or 46% and the none group, 88/214 or 41%). However, no significant difference in the estimated median time to new BMs could be observed (*p* = 0.357, Figure 3E). 

At last follow-up, the vast majority of TT group patients (58/59 or 98%) presented with stable or decreased BMs. There was a trend towards a longer estimated time to progression in patients with TT as compared with patients in the IT group (*p* = 0.058, Figure 3F) and as compared with the none group (*p* = 0.054). There was no statistically significant difference in the local PFS in patients with TT at GKRS1 (*n* = 35) as compared with patients who received TT after GKRS1 (*n* = 24). 

The occurrence of radiation reaction in patients who received concomitant TT at GKRS1, also did not show any differences as compared with patients without IT or TT (*p* = 0.143) or patients whose TT was started > 30 days after GKRS (*p* = 0.715). In a similar fashion, the occurrence of radiation necrosis in patients who received concomitant TT at GKRS1, also did not show any differences as compared with patients without IT or TT (*p* = 0.741) or patients whose TT was started > 30 days after GKRS (*p* = 0.180). These analyses did not differ even when the occurrence of RR and RN were pooled (*p* = 0.354 at GKRS1 and *p* = 0.242 after GKRS1). The Kaplan–Meier estimated mean time to occurrence of RR alone, RN alone, and RR/RN pooled did not show any significant differences in patients, who received TT at GKRS1 versus those patients who received TT after GKRS1 (*p* = 0.397 for RR, *p* = 0.171 for RN, and *p* = 0.705 for RR/RN pooled).

## 3. Discussion

### 3.1. Survival after GKRS in Relation to IT or TT

So far, most of the clinical literature on radiosurgery and IT or TT has been derived from melanoma BM, while data on NSCLC are scarce [8,9,10]. We present data on a representative real-life cohort of 496 NSCLC patients with BMs, who were treated with GKRS in combination with IT or TT as compared with patients who did not receive IT or TT. 

Oncological therapies in our patients resembled the typical frequency distribution of driver oncogene mutations for NSCLC patients [11]. Molecular targets are found in up to 69% of NSCLC [11]. According to the ESMO guidelines published in 2018, the treatment option for those patients without an actionable oncogenic driver is still limited to platinum-based doublet therapy [1]. The epidermal growth factor receptor (EGFR)-activating mutations are the most common targetable mutations, which were also observed in our study population. Our study confirmed that in NSCLC patients, programmed cell death-1 (PD-1) targets were most commonly expressed [12,13]. 

As expected, patients with better baseline prognostic scores presented with better overall survival. Until recently, the survival of BM patients has been estimated by different scores based on clinical or radiological baseline characteristics such as the GPA, RPA, and SIR [14,15,16]. However, the validity of these scores has been criticized before, due to the lack of other influencing factors, such as gene alterations [1,17]. Therefore, a novel disease- and molecular-specific prognostic score, the Lung-molGPA, has recently been evaluated for its prognostic value in overall survival [1,17]. Indeed, in our real-life cohort, the survival after BM diagnosis was significantly longer than calculated prognostic GPA, RPA, and SIR scores among all patients and was in the range of the Lung-molGPA score for patients with IT or TT but not in the none group.

Few existing studies on NSCLC BM patients reported controversial results on survival after SRS and progression-free survival (PFS) in relation to IT or TT. While in some studies no significant differences in survival could be observed [9,18], others reported a favorable survival outcome in patients treated with IT or TT [6,19,20]. However, most of these studies, so far, have reported on rather small NSCLC patient cohorts or combined patients of different primary tumors. In a recent study from Singh SA et al., the beneficial effect of SRS and concurrent IT or TT on PFS was observed in 99 NSCLC BM patients [21]. In contrast, a recent study by Singh C et al. evaluated 85 NSCLC BM patients and found no significant benefit for patients undergoing SRS and IT [18].

In our study, survival after GKRS1 was significantly longer between pooled IT or TT as compared with the none group and also among the separately analyzed IT or TT treatment groups. Nevertheless, the outcome of oncological patients can be severely biased by different baseline characteristics and small group sizes. Therefore, comparisons of larger patient groups with similar baseline values are essential [9]. Consequently, we performed several subanalyses adjusting for KPS, sex, multiple BMs, and RPA class. Nevertheless, the clear benefit in survival after GKRS with concurrent IT or TT treatment remained significant even after these adjustments.

### 3.2. Local and Distant Cerebral Tumor Control after GKRS in Relation to IT or TT

Recent studies of BM patients with different primary tumor origins observed no significant differences in local PFS in patients with or without IT [6,9,18]. In our cohort, the timing of concomitant GKRS and IT or TT seems to affect local progression-free survival after GKRS. Overall, our local PFS did not differ between patients with and without IT or TT. However, in patients with IT or TT at GKRS1, PFS was significantly longer than in patients with IT or TT after GKRS1. These findings did not remain statistically significant when analyzing patients with IT or TT separately, but this was most likely due to smaller patient numbers, since a clear trend was seen, especially among patients with TT.

These results could be explained by the beneficial aspects of EGFR mutations and their therapeutic agents. Previous studies have reported that patients with EGFR mutations have better therapy responses and longer intracranial PFS after radiotherapy [20,22]. EGFR-targeting therapies, such as Erlotinib and Gefitinib, might thereby lead to longer PFS and delayed progression of the intracranial disease [20]. The most commonly used TT in our study were the first-generation EGFR-targeting therapy types, Erlotinib and Gefitinib. Recent studies have also shown a better PFS in second or third generations, such as Afatinib or Osimertinib as compared with first generations, due to their capability to cross the blood-brain barrier [23]. In our study, these differences were not observed, possibly due to the smaller sample size of third generation therapy.

Despite the favorable local tumor control of BMs after GKRS, the occurrence of new BMs is a well-known confounder in NSCLC patients. Therefore, metastatic NSCLC patients should be clinically and radiologically followed in tight time frames, regardless of the selected therapy [10]. About half of all our patients developed new distant BMs after GKRS1. Among these patients, the majority were treated with another GKRS. Of note, the median time to the occurrence of new BMs did not differ between the four treatment groups, although the percentages of patients with new BMs seemed to be higher in the IT or TT group as compared with the none group. This could be well explained by the fact that patients in the IT or TT group had a significantly longer survival, and thus a longer follow-up time after GKRS1. Moreover, as Reynders et al. reported, the abscopal effect is thought to take up to several weeks or even months before clinical response can be observed [24]. 

### 3.3. Complications after GKRS in Relation to IT or TT

Overall, complication rates in our study were in the lower range of previously reported rates prior to the era of IT or TT [25]. Data on the specific toxicity in NSCLC BM patients, treated with combined radiosurgery and IT or TT, are still scarce but concerns of increased neurotoxicity have been raised [26]. A recent study of 180 patients of heterogeneous primary tumors reported an increased risk of radiation necrosis (RN) in patients who received IT [27]. 

In contrast, Shepard et al. reported no increased rates of RN or intratumoral hemorrhage as compared 17 NSCLC-BM patients with and 34 patients without concurrent IT [9]. The exact mechanism and extent of how concurrent oncological therapies may influence RN is still unknown [27]. Nevertheless, any inflammatory response of the brain to high-dose radiation could potentially cause RN. However, the occurrence of RN is also influenced by other known risk factors such as primary tumor histology, BM diameter, and history of previous radiation treatments [25,27]. In our study, the rates of adverse reactions in concomitant use with IT or TT at and after GRKS1 were not statistically distinguishable as compared with patients without IT or TT. However, concomitant IT or TT at GKRS1 seemed to be linked with a lower risk of developing RR/RN as compared with those patients with IT or TT after GKRS1 or patients without IT or TT at all. 

### 3.4. The Role of Radiosurgery in the Era of IT or TT

Since the effect of systemic therapies is often limited due to restricted access through the blood-brain barrier, local treatment options for patient with BMs are essential [28]. Stereotactic radiosurgery results in higher tumor control rates and almost no neurocognitive deterioration as compared with WBRT [3,4]. 

Preclinical studies have shown that the combination of radiotherapy and immunotherapy improved the adaptive antitumor immunity as a mediator of systemic effects [24]. After radiation, the tumor environment changes, causing the release of tumor antigens, and therefore increases the antitumor effect of immunotherapy [28]. Thus, closer timing of concurrent radiosurgery and oncological therapies may increase the synergic effect of both treatments, leading to better brain control and maybe even an improved systemic response via the abscopal effect [24,29]. One recent study of heterogeneous primary tumors found that patients with concurrent IT and radiosurgery presented with a decreased likelihood of developing more than three new BMs after radiosurgery as compared with patients without concurrent IT [6]. However, almost half of our patients in all treatment groups developed new BMs after GKRS1, leading to further GKRS treatments, thus, highlighting the importance of radiosurgery in modern BM treatment. 

Moreover, as described above, the timing of concomitant GKRS and IT or TT seems to play a crucial role in local progression-free survival after GKRS1. In contrast, in our cohort, survival among patients with IT or TT is better as compared with the none group, regardless of the timing of IT or TT. However, 45% of our patients that received IT or TT after GKRS1 did undergo multiple GKRS treatments. Consequently, treatment with IT or TT per se, and also the concurrent timing in regard to the radiosurgery treatment seems to be beneficial for NSCLC patients with BMs. 

However, given these findings, further prospective studies are necessary to verify the optimal timing of GKRS and concurrent IT or TT.

## 4. Materials and Methods 

### 4.1. Patient Sample and Data Evaluation

This study complied with the Declaration of Helsinki and was approved by the local ethics review committee of the Medical University of Vienna (EK 1949/2018). At our department, patients have been treated with GKRS since 1992. Since the implementation of the new Gamma Knife^®^ Perfexion^TM^ in 2012, 496 NSCLC patients with BMs were treated between 2012 and 2018. Data were censored as of October 2019.

Twelve patients (12/496, 2%) had their first GKRS prior to 2012 but underwent repeat GKRS between 2012 and 2018, and thus were included in our study. All patients with the diagnosis of BM in NSCLC patients, an age > 18 years, and at least one GKRS treatment for at least one BM were retrospectively included in our study. As shown in Table 1, the KPS range of our patients varies, which is explained by our treatment policy, i.e., we are able to treat even palliative patients. 

To evaluate radiosurgery treatment in relation to the oncological therapies, data on the treatment with IT or TT were reviewed at the time of radiosurgery treatment (±30 days) and after GKRS1 (>30 days) [5,30]. Patients were grouped accordingly (Table 2).

### 4.2. Radiosurgery Technique

At our institution, patients were planned with the Leksell GammaPlan^®^ (Stockholm, Sweden), based on the planning sequences, performed on a 1.5 Tesla magnet MRI with Gadolinium contrast-enhanced T1-weighted MRI sequences in axial and coronal planes. Multiplanar T2-weighted MRI sequences were additionally performed as appropriate. The Leksell Gamma Knife^®^ Perfexion^TM^ (Elekta AB, Stockholm, Sweden) was used for the treatment. All metastases, defined as a contrast-enhanced tumor mass on T1 sequences, visualized on planning sequences were treated. The whole lesion was covered without an additional margin. 

Overall, 1870 BMs were treated in 717 radiosurgery procedures, with a median number of two (1–15) BMs per GKRS treatment including all GKRS treatments. Table 1 depicts details on GKRS parameter for the first GKRS treatment. The median time between initial BM diagnosis and GKRS1 was 0.6 months (0.0–32.5). 

The majority of patients (343/496, 69%) underwent one GKRS, while 153/496 (31%) patients received multiple treatments due to newly diagnosed BMs or two-fraction dose-staged GKRS, as described before [31]. Patients with IT or TT more often suffered from multiple BMs at GKRS1 than patients in the none group (*p* = 0.020). Consequently, patients with IT or TT more often underwent GKRS with a boost dose (17%), a reduced dose due to multiple BMs as compared with patients in the none group (13%, *p* = 0.010). Overall, BMs were planned on the 50% (IT or TT: 50, 40–90%; none: 50, 40–90%) isodose line, with a median prescription dose of 18 Gy (IT or TT: 18, 10–20 Gy; none: 18, 8–20 Gy), a median central dose of 30 Gy (IT or TT: 30, 13–45 Gy; none: 34, 16–44 Gy), and a median treatment volume of 0.6 cm^3^ (IT or TT: 0.6, 0.1–13.8 cm^3^; none: 0.8, 0.1–27.7 cm^3^). 

### 4.3. Follow-Up and Outcome Evaluation

Patients were routinely followed in three-month intervals. Patients who were lost to follow-up were included in the study but excluded from the outcome analysis (Figure 1). All complications after radiosurgery were evaluated by the study investigating neuro-radiologist on follow-up MRIs, blinded to the clinical follow-up data. Progression was defined according to the RANO criteria [32]. Radiation reaction, radiation necrosis, and intralesional hemorrhage were defined as previously described [33,34,35]. Intracranial progression-free survival was defined as the absence of local control failure of known BMs following initial radiosurgery therapy.

### 4.4. Statistical Analysis

Categorical data were presented as counts and percentages, and continuous parameters as median and range. The Chi-square test was used to analyze the counts. Bonferroni–Holm correction was used for multiple comparisons as appropriate. The Kaplan–Meier method was used to estimate survival, time to local progression, the occurrence of adverse reactions, or new BMs. Differences between groups were compared with the generalized Wilcoxon–Breslow test or log-rank test as appropriate. For all statistical calculations, *p*-values < 0.05 were considered to be statistically significant. Statistical analyses were carried out with IBM SPSS Statistics for Windows (Version 24 Armonk, IBM Corp., New York, NY, USA).

## 5. Conclusions

In conclusion, the concomitant use of GKRS and systemic treatment with IT or TT for NSCLC patients with BMs showed an increase in overall survival, without increased complications related to GKRS. Therefore, the combined treatment of GKRS and IT or TT seems to be a safe and powerful treatment option and emphasizes the role of radiosurgery in modern BM treatment.

## Figures and Tables

**Table 1:** Characteristics of the study population.

	Time of First GKRS, Total Sample (*n* = 496)	Patients with IT or TT at or after GKRS1 “IT or TT Group” (*n* = 186 *)	Patients without IT or TT at or after GKRS1 “None Group” (*n* = 286 *)	IT or TT vs. None Group
Age	64	62	65	*p* = 0.004
in years, median (range)	(28–87)	(28–87)	(36–87)	
Female/male ratio	258:238	102:84	141:145	*p* = 0.239
KPS	80	80	80	*p* = 0.001
in %, median (range)	(40–100)	(40–100)	(40–90)	
KPS groups				*p* = 0.003
≥80%	345 (70%)	143 (77%)	183 (64%)	
<80%	151 (30%)	43 (23%)	103 (36%)	
ECM status at time of BM diagnosis				*p* = 0.019
Yes	321(65%)	134 (72%)	176 (62%)	
No	175 (35%)	52 (28%)	110 (38%)	
NSCLC subtype				*p* = 0.020
Adenocarcinoma	416 (84%)	169 (91%)	225 (79%)	
Squamous cell carcinoma	66 (13%)	13 (7%)	51 (18%)	
Large cell neuroendocrine carcinoma	7 (2%)	2 (1%)	5 (2%)	
Large cell carcinoma	5 (1%)	2 (1%)	3 (1%)	
Adenosquamous carcinoma	1 (0%)	0 (0%)	1 (0%)	
Polymorphous carcinoma	1 (0%)	0 (0%)	1 (0%)	
CNS treatment before GKRS1				*p* = 0.637
None	415 (84%)	159 (85%)	239 (83%)	
WBRT and/or fRT	36 (7%)	15 (9%)	20 (7%)	
BM resection without RT	20 (4%)	6 (3%)	11 (4%)	
BM resection with WBRT and/or fRT	25 (5%)	6 (3%)	16 (6%)	
Localization of BM at initial diagnosis				*p* = 0.861
Multiple	276 (56%)	112 (60%)	150 (52%)	
Frontal	58 (12%)	20 (11%)	36 (13%)	
Parietal	22 (4%)	7 (4%)	14 (5%)	
Temporal	23 (5%)	8 (4%)	15 (5%)	
Occipital	35 (7%)	11 (6%)	22 (8%)	
Central	29 (6%)	9 (5%)	19 (7%)	
Basal ganglia, brainstem, other	11 (2%)	4 (2%)	7 (2%)	
Cerebellar	42 (8%)	15 (8%)	23 (8%)	
Predicted survival after prognostic scores				
in months, median (range)				
GPA general	3.8 (2.6–11.0)	3.8 (2.6–11.0)	3.8 (2.6–11.0)	*p* = 0.409
GPA specific	5.5 (3.0–14.8)	5.5 (3.0–14.8)	5.5 (3.0–14.8)	*p* = 0.450
RPA	4.5 (2.3–7.7)	4.5 (2.3–7.7)	4.5 (2.3–7.7)	*p* = 0.374
SIR	6.0 (2.1–8.8)	6.0 (2.1–8.8)	6.0 (2.1–8.8)	*p* = 0.009
Lung-molGPA for adenocarcinoma	13.7 (6.9–46.8)	13.7 (6.9–46.8)	13.7 (6.9–46.8)	*p* = 0.004
Lung-molGPA for nonadenocarcinoma	9.8 (5.3–12.8)	9.8 (5.3–12.8)	9.8 (5.3–12.8)	*p* = 0.126
Parameters of first GKRS				
Number of treated BMs, median (range)	2 (1–13)	2 (1–9)	2 (1–13)	*p* = 0.001
Treatment volume in cm^3^, median (range)	0.6 (0.1–27.7)	0.8 (0.1–27.7)	0.6 (0.1–13.8)	*p* < 0.001
Isodose in %, median (range)	50 (40–90)	50 (40–90)	50 (40–90)	*p* < 0.001
Prescription dose in Gy, median (range)	18 (8–20)	18 (8–20)	18 (10–20)	*p* = 0.010
Central dose in Gy, median (range)	32 (13–45)	34 (16–44)	30 (13–45)	*p* < 0.001

BM, brain metastasis; CNS, central nervous synstem; ECM, extracranial metastases; fRT fractionated radiotherapy; GKRS, Gamma Knife Radiosurgery; GPA, graded prognostic assessment; IT, immunotherapy; KPS, Karnofsky performance status scale; RPA, recursive partitioning analysis; SIR, Score Index for Radiosurgery; TT, targeted therapy; WBRT, whole brain radiation therapy. (*) After excluding these patients, the baseline characteristics were additionally evaluated for 186/472 (39%) patients in the “IT or TT group” (defined as IT or TT or a combination of IT and TT) and for 286/472 (61%) patients in the “none group” (defined as no IT or TT).

**Table 2:** Immunotherapy (IT) or targeted therapy (TT) at or after the first Gamma Knife radiosurgery treatment (GKRS1) in NSCLC patients.

Treatment Group (*n* = 488)	Number of Patients (%)
**IT group** (90/488, 18%)	**90**
Nivolumab ^1^	38 (42%)
Pembrolizumab ^1^	29 (32%)
Atezolizumab ^2^	15 (17%)
Durvalumab ^2^	5 (6%)
Unknown therapy types, only documented as IT (external study patients)	3 (3%)
**TT group** (72/488, 15%)	**72**
Erlotinib ^3^	14 (19%)
Gefitinib ^3^	11 (15%)
Afatinib ^3^	11 (15%)
Alectinib ^4^	4 (6%)
Crizotinib ^5^	4 (6%)
Osimertinib ^3^	3 (4%)
Nintedanib ^6^	3 (4%)
Brigatinib ^4^	2 (3%)
Ceritinib ^4^	1 (1%)
Osimertinib and Afatinib	9 (13%)
Combinations of TT	10 (14%)
**Other/IT and TT combination group** (24/488, 5%)	**24**
Multiple combinations	20 (83%)
Bevacizumab	4 (17%)
**None group** (286/488, 59%)	286
No data on IT or TT (16/488, 3%)	16

^1^ PD-1, programmed cell death-1 inhibitor; ^2^ PD-L1, programmed death ligand-1 inhibitor; ^3^ EGFR, epidermal growth factor receptor; ^4^ ALK, anaplastic lymphoma kinase; ^5^ ALK + ROS1, c-ros oncogene 1 inhibitor; ^6^ VEGFR, vascular endothelial GFR inhibitor; FGFR, fibroblast GFR inhibitor; PDGFR, platelet-derived GFR inhibitor. GKRS, Gamma Knife radiosurgery; IT, immunotherapy; NSCLC, non-small cell lung cancer; TT, targeted therapy. Bold: to highlight the treatment groups.

**Table 3:** Radiologically diagnosed intralesional hemorrhage or radiation reaction/necrosis after GKRS1 in relation to IT or TT.

IT/TT at or after GKRS1 (*n*)	Intralesional Hemorrhage after GKRS1 (*n* = 379)		Radiation Reaction after GKRS1 (*n* = 379)		Radiation Necrosis after GKRS1 (*n* = 379)	
	Yes, *n* (%)	No, *n* (%)	Yes, *n* (%)	No, *n* (%)	Yes, *n* (%)	No, *n* (%)
IT (*n* = 82)	0	82 (100)	9 (11)	73 (89)	8 (10)	74 (90)
TT (*n* = 59)	0	59 (100)	3 (5)	56 (95)	3 (5)	56 (95)
Other/Combination (*n* = 24)	0	24 (100)	1 (4)	23 (96)	2 (8)	22 (92)
None (*n* = 214)	2 (1)	212 (99)	23 (11)	191 (89)	15 (7)	199 (93)
Total (*n* = 379)	2 (1)	377 (99)	36 (9)	343 (91)	28 (7)	351 (93)

BM, brain metastases; GKRS, Gamma Knife radiosurgery; IT, immunotherapy; MRI, magnetic resonance imaging; TT, targeted therapy.
